# Psychological Distress Among Occupational Health Professionals During Coronavirus Disease 2019 Pandemic in Spain: Description and Effect of Work Engagement and Work Environment

**DOI:** 10.3389/fpsyg.2021.765169

**Published:** 2021-12-16

**Authors:** Carlos Ruiz-Frutos, Mónica Ortega-Moreno, Guillermo Soriano-Tarín, Macarena Romero-Martín, Regina Allande-Cussó, Juan Luis Cabanillas-Moruno, Juan Gómez-Salgado

**Affiliations:** ^1^Department of Sociology, Social Work and Public Health, Faculty of Labor Sciences, University of Huelva, Huelva, Spain; ^2^Safety and Health Postgraduate Program, Universidad Espíritu Santo, Guayaquil, Ecuador; ^3^Department of Economy, Faculty of Labor Sciences, University of Huelva, Huelva, Spain; ^4^Asociación Española de Medicina del Trabajo – Spanish Association of Specialists in Occupational Health Medicine, Valencia, Spain; ^5^Department of Nursing, Faculty of Nursing, University of Huelva, Huelva, Spain; ^6^Department of Nursing, Universidad de Sevilla, Seville, Spain; ^7^Department of Preventive Medicine and Public Health, Universidad de Sevilla, Seville, Spain

**Keywords:** COVID-19, psychological distress, work engagement, occupational health, healthcare professionals, occupational medicine, nursing

## Abstract

The impact of the coronavirus disease 2019 (COVID-19) pandemic on the mental health of hospital health professionals has been widely described, but few studies have focused on occupational health professionals. Therefore, the objective of this study was to assess psychological distress (PD) of occupational health workers and its relationship with their work engagement (WE) and work environment characteristics. A cross-sectional survey was conducted. A sample of 499 nurses and physicians participated in the study. Variables included demographic data, work environment characteristics, work engagement Utrecht Work Engagement Scale (UWES-9) and psychological distress General Health Questionnaire (GHQ-12). The Chi-square Automatic Interaction Detection method was performed for data analysis. Data collection took place *via* the internet between April 23 and June 24, 2020. A total of 65.53% of the participants had PD, and the total mean score of the UWES-9 scale was 34.80 (SD = 10.69). Workload, conflicts, stressful situations, and less job satisfaction were significantly related to a higher percentage of PD (*p* < 0.05). Participants with low engagement showed higher levels of PD (76.7%; *p* < 0.001). The dedication was revealed as the most significant dimension. Interventions aimed at promoting resilience and coping strategies are suggested. WE should be fostered as a preventive measure against PD among occupational health workers. By protecting workers, occupational health departments have a shared responsibility with public health in containing the pandemic. Therefore, it is essential to prevent the psychological impact that this responsibility may have on occupational health workers by implementing prevention measures.

## Introduction

The health crisis caused by the coronavirus disease (COVID-19) has been considered an unprecedented global pandemic that threatens the entire world. Public health interventions have been implemented to minimize the negative effect of the pandemic on the physical and mental health of the population (Pan et al., 2020). Regarding physical health, the symptoms and prognosis of COVID-19 patients are remarkably diverse. Symptoms and signs present with unpredictable intensity, ranging from asymptomatic to severely compromised, leading to death (Adhikari et al., 2020). Due to this variability, in addition to the unavailability of a specific treatment against the virus, the most effective approach to protecting the population is preventive measures to avoid exposure to the virus and vaccination (Singhal, 2020).

Regarding mental health, the evidence suggests that anxiety, depression, and stress are the common and expectable reactions to the COVID-19 pandemic (Rajkumar, 2020). Health professionals are a particularly vulnerable group to psychological distress (PD), due to their level of exposure and the nature of their work (Chew et al., 2020). Previous studies have described the psychological impact of the pandemic on healthcare professionals, resulting in anxiety and depression (Suryavanshi et al., 2020; Xiang et al., 2020; da Silva and Neto, 2021), insomnia (Pappa et al., 2020; da Silva and Neto, 2021), post-traumatic stress (Preti et al., 2020), and physical and mental exhaustion or emotional disorders (Kang et al., 2020). Health professionals under quarantine, those who worked caring for patients with COVID-19, or those who had relatives or friends infected by the virus developed considerably more anxiety, depression, frustration, fear, and post-traumatic stress than those who had not been subjected to these conditions (Xiang et al., 2020). The risk of PD increases when working directly with patients suffering from COVID-19, due to fear of their own contagion and concern for the health of their relatives (Babicki et al., 2021). This entails the need to ensure adherence of healthcare professionals to appropriate infection prevention measures during the health crisis (Temsah et al., 2020).

In this scenario, occupational health faces a demanding challenge. Although COVID-19 has been accepted as a public health problem, it is less common to consider it an occupational disease (Koh and Goh, 2020). Although vaccination is the most successful strategy to prevent the spread of the virus and the actual vaccines are highly effective, herd immunity is required to end the pandemic. In Europe, the complete vaccination figures do not reach the desired threshold to control the pandemic safely, so it is recommendable to maintain other preventive measures such as social distancing, face masks, and hand hygiene (Cihan, 2021). Every job involving contact with the public and physical proximity is subject to the risk of exposure, given the high incidence of the disease. This is a burden for this sector since, in addition to the risks inherent in each job, there is also the risk of being infected for many workers (Burdorf et al., 2020). The pandemic has required Spanish institutions to develop procedures facing the exposure to the new coronavirus and during the alarm state, those workers who required periods of isolation or who had been infected were in a situation considered as an occupational accident (Government of Spain, 2020; Instituto Nacional de Seguridad y Salud en el Trabajo, 2020). In March, during the coronavirus outbreak, sick leaves increased 116% in Spain, and the figures were particularly high among healthcare workers (457%) (Calvo-Bonacho et al., 2020). This measure has forced occupational health professionals to work in harsh conditions, under pressure, with an increase in their workload, and with wider schedules and limited human resources (Santarone et al., 2020). Authors have pointed out the relevant role of occupational health in the management of the response to the crisis, providing safety, surveillance, and health protection to the workers under their care (Fadel et al., 2020). Also, once the worst effects of the pandemic have been overcome, it is proposed to reactivate the economy, for which many workers must return to their jobs safely, and thus, the occupational health professionals should adapt their work environment to the new normality conditions (Rueda-Garrido et al., 2020). Therefore, occupational health workers, in addition to the risk of PD associated with being health workers, are under pressure to provide safe working conditions for workers and to prevent the spread of the virus in the workplace.

Maintaining the psycho-emotional wellbeing of health workers and fostering their resilience are crucial in addressing and containing COVID-19 (Chen et al., 2020). It has been proposed that institutions should implement training and confidence in prevention equipment and measures (Preti et al., 2020) and interventions aimed at creating a psychologically safe environment, sound leadership, clear organizational strategies, and meaningful support for the team (Blake et al., 2020). Work engagement (WE) could help professionals cope with work-related PD and contribute to their wellbeing and health (Malagón-Aguilera et al., 2019). Work engagement is a positive and satisfactory attitude related to work, characterized by vigor, dedication, and total absorption and concentration in the activity. Vigor refers to high levels of energy, persistence, and mental endurance. Dedication refers to being strongly involved in his/her work and experiencing a sense of importance and enthusiasm. Absorption refers to being fully concentrated and happily absorbed at work (Schaufeli et al., 2002). Work engagement is considered as one of the constructs of wellbeing, a way to reduce the prevalence of stress or burnout among healthcare workers from a positive organizational psychological perspective, and as an indicator of intrinsic motivation for work (García-Renedo et al., 2006).

The work environment plays an important role in the development of WE. It has been described how transformational leadership, structural empowerment, a positive work climate, and social support enhance WE (García-Sierra et al., 2016). In addition, the job characteristics, such as skill variety, task identity, task significance, autonomy, and job feedback, promote the feelings of WE among workers (Wan et al., 2018). Work engagement is fostered by labor resources (e.g., autonomy and social support by peers or higher professional roles) and recovery provided by emotional contagion outside of work, as well as personal resources, such as self-efficiency, or belief in the own ability to perform the job appropriately (García-Renedo et al., 2006). In contrast, workload and overtime work have a negative effect on workers and reduce their WE capacity (Ando and Kawano, 2018). The distress generated by the frustration of being forced to adopt behaviors that are not considered morally acceptable also negatively influences WE (Lawrence, 2011).

Therefore, since WE fosters a positive attitude toward work and brings personal benefits for workers such as job satisfaction, decreased burnout, work effectiveness, and wellbeing, it could be a valuable resource to address the psychological impact of the pandemic (Lawrence, 2011; García-Sierra et al., 2016; Keyko et al., 2016). Both WE and a favorable work environment promote feelings of wellbeing in workers, positive toward work, and a desire to stay (Wan et al., 2018).

For all the above, the hypothesis proposed by this study is whether sociodemographic variables, work environment, and WE influence the level of PD of occupational health professionals. Many studies have centered on the impact of the COVID-19 pandemic on the mental health of healthcare professionals (Chew et al., 2020; Kang et al., 2020; Pappa et al., 2020; Preti et al., 2020; da Silva and Neto, 2021), but few studies have focused on the occupational health professionals. This study aimed to describe the impact of the COVID-19 pandemic on psychological welfare among the occupational health professionals in Spain, during the pandemic outbreak. Our objective was to assess the PD of occupational health workers and its relationship with their WE and work environment characteristics.

## Materials and Methods

### Design

A cross-sectional study was conducted.

### Participants

The study population was the occupational health professionals actively working during the first wave of the COVID-19 pandemic in Spain. As the inclusion criteria, it was established physicians or nurses working in an occupational health department during the first stage of the COVID-19 pandemic. Those professionals who did not live in Spain at the time of the study or were not actively working (sick leave, unemployed, or retired) were excluded.

Participants were recruited from the Spanish Association of Occupational Medicine (1,300 members) and the Spanish Association of Occupational Health Nursing (250 members) as they are the primary associations of occupational health in Spain. Considering a confidence level of 95%, a precision of 4.5%, and an adjustment for losses of 10%, an optimal sample size of 427 participants was estimated for our study population. After data collection, the sample was over the estimated size with 499 participants, 402 (30.9%) doctors, and 97 (38.8%) nurses.

### Instruments

#### Sociodemographic and Work Environment Variables

Data regarding the sociodemographic and work environment variables were collected by an *ad hoc* questionnaire made for this purpose. The questionnaire included socio-labor variables such as sex, age, educational level (degree, master’s, or Ph.D.), marital status, children, pets, type of housing (with or without outdoor space), teleworking, work center (public or private), and professional profile (nurse or physician). It also included variables related to the work environment such as perception of conflict, perception of safety, acceptance of risk, workload, perceived stress, and job satisfaction. The categorization of variables related to the work environment, with scores between 1 and 10, was transformed into the negative response for values less than or equal to 5 and the positive response otherwise.

#### General Health Questionnaire

The General Health Questionnaire (GHQ-12) (Goldberg et al., 1997) on its Spanish adapted version (Sánchez-López and Dresch, 2008) was used to measure PD. Each item had four response options, scoring 0 points with options 1 or 2, or 1 point with options 3 or 4. As the questionnaire has 12 items, the overall score ranged from 0 to 12. In this study, a cutoff point of 3 was agreed, with scores equal to or greater than 3 considered as signs of PD (Jackson, 2006). The internal consistency of the instrument has been previously tested, with Cronbach’s index ranging from α = 0.76 to α = 0.82 for the Spanish population (Sánchez-López and Dresch, 2008; Padrón et al., 2012).

#### Utrecht Work Engagement Scale

The WE was measured by Utrecht Work Engagement Scale (UWES-9) in its short version (Schaufeli et al., 2006). It is an instrument designed to be self-managed and features nine items, with Likert-type response options ranging from 0 (Never) to 6 (Always). It consists of three dimensions, namely, vigor, dedication, and absorption. The score was calculated in each dimension, adding the items in each dimension and dividing the result by the number of items that make up each dimension. Scores obtained in each dimension of the UWES-9 were categorized, distinguishing between low, intermediate, or high categories, where the “low” category grouped participants who scored between the minimum and the 25th percentile, “intermediate” category grouped those who were scoring 50%, and “high” category grouped those who scored between the 75th percentile and the maximum value. The Spanish version of the instrument has achieved the following Cronbach’s internal consistency indexes: vigor (α = 0.82), dedication (α = 0.86), and absorption (α = 0.8) (García-Iglesias et al., 2021).

### Procedure

The questionnaire was distributed online among the Spanish Association of Occupational Medicine and the Spanish Association of Occupational Health Nursing. All members who had given their consent to be contacted for research purposes were sent an invitation to participate *via* email, and a reminder. A link to the survey was also available at both Association websites. Participants were informed about the purpose and conditions of the study at the beginning of the questionnaire. Through informed consent, the participants voluntarily expressed their desire to participate in the study. Participants were free to leave the study at any time, as unfinished questionnaires were not included in the database. The anonymity and confidentiality of the data collected were maintained.

The online survey platform Qualtrics^®^ was used for data collection and storage. The survey was to be completed from any electronic device (i.e., tablet, laptop, and mobile phone) with internet access. Data collection took place between April 23 and June 24, 2020 (the state of alarm began on March 13).

### Statistical Analysis

Absolute frequencies and percentages of the variables collecting socio-labor information (i.e., sex, age, educational level, marital status, children, pets, type of housing, teleworking, work center, and professional profile) were calculated, as well as for those variables collecting data regarding the work environment (i.e., perception of conflict, perception of safety, acceptance of risk, workload, perceived stress, and job satisfaction). Work Engagement dimensions were evaluated by the median score. To contrast whether there is a relationship between these variables and the presence of PD, the χ^2^ test of independence was performed; also, the Chi-square Automatic Interaction Detection (CHAID) method determined which variables played a remarkable role, choosing those predictors with lower adjusted *p*, as long as that value was less than or equal to the significance level set to *p* = 0.05. The CHAID method (Kass, 1980) is a hierarchical classification tool that determines which factors or predictors are most related to the classification criterion, using the chi-square test of independence and selecting the factor that has the smallest *p*-value, i.e., the most significant factor. The sample was then divided according to the levels of the chosen factor, and the same criterion was applied to each resulting group, dividing again repeatedly until it was not possible to continue dividing or no other significant factor was found (α = 0.05). The level of significance to merge the two categories of a predictor and to divide a node by the most significant predictor was, in both cases, 0.05. The analyses were carried out with the statistical software SPSS 26.0© SPSS: (IBM Corporation, 2019) and R, version 4.0.0© R: (Fox and Bouchet-Valat, 2020).

### Ethical Considerations

This study has the favorable report of the Research Ethics Committee of Huelva, belonging to the Andalusian Ministry of Health (PI 036/20), having complied with all the ethical principles contained in the Declaration of Helsinki.

## Results

### Socio-Labor Variables and Psychological Distress

A total of 65.53% of the participants had PD (GHQ ≥ 3), with a similar percentage in occupational health physicians (65.67%) and occupational health nurses (64.95%). The sample is mostly composed of women (65.73%), and 50% were over 51 years old. Regarding the level of education, 55.51% had a postgraduate, MSc, or Ph.D. degree. Most of them (78.16%) were married or lived with a couple, 79.56% had children, 33.67% claimed to have pets, and 76.75% had a house with outdoor space. Neither of these variables influenced having higher PD. With respect to the type of work center, the highest percentage (63.33%) worked for a private company and the remaining 36.67% for the public sector. PD levels of older public sector workers were higher (72.68%) than those of private company workers (61.39%; *p* = 0.011) (Table 1).

**TABLE 1:** Socio-labor variables and psychological distress (PD).

	Total		GHQ < 3		GHQ ≥ 3			
	Cases (N) 499	% of total 100%	Cases (N) 172	% line 34,47%	Cases (N) 327	% line 65,53%	Chi-square statistic χ^2^	*p*-Value *p*
**Sex**								
Male	171	34.27%	83	48.54%	88	51.46%	22.80	< 0.001
Female	328	65.73%	89	27.13%	239	72.87%		
Age*								
51 or younger	249	50.00%	74	29.72%	175	70.28%	5.12	0.024
Older than 51	249	50.00%	98	39.36%	151	60.64%		
**Educational level**								
University degree	222	44.49%	69	31.08%	153	68.92%	2.03	0.154
Postgraduate: MSc or Ph.D.	277	55.51%	103	37.18%	174	62.82%		
**Marital status**								
With a partner	390	78.16%	142	36.41%	248	63.59%	2.98	0.084
Without a partner	109	21.84%	30	27.52%	79	72.48%		
**Children**								
No	102	20.44%	32	31.37%	70	68.63%	0.54	0.461
Yes	397	79.56%	140	35.26%	257	64.74%		
**Pet**								
No	331	66.33%	111	33.53%	220	66.47%	0.38	0.538
Yes	168	33.67%	61	36.31%	107	63.69%		
**Type of housing**								
Without outdoor space	116	23.25%	40	34.48%	76	65.52%	0.00	0.997
With outdoor space	383	76.75%	132	34.46%	251	65.54%		
You work*								
From home	117	26.23%	85	72.65%	32	27.35%	5.14	0.077
Away from home	312	69.96%	191	61.22%	121	38.78%		
Both from and away from home	17	3.81%	12	70.59%	5	29.41%		
**Work center**								
Public	183	36.67%	50	27.32%	133	72.68%	6.53	0.011
Private or associated	316	63.33%	122	38.61%	194	61.39%		
**Profile**								
Occupational health nurse	97	19.44%	34	35.05%	63	64.95%	0.02	0.893
Occupational medicine	402	80.56%	138	34.33%	264	65.67%		
**Active**								
Yes	499	1.00%	172	34.47%	327	65.53%		

*Total cases do not correspond because the information is not collected in all subjects.

### Work Environment and Psychological Distress

Half of the participating professionals (50.22%) claimed that there was labor conflict in the company, presenting a higher percentage of PD for those participants working in settings where that conflict existed (76.34%) than those for which this conflict did not exist (52.70%; *p* < 0.001). The presence of risk was perceived by 83.63% of workers, and the risk of contracting the disease was accepted by 66.82%, without having the statistical significance of the ones with the highest PD as compared to those who claimed not to be. The higher workload for 91.48% of workers was related to a higher percentage of PD among them (67.16%) than among those who claimed not to have a higher workload (36.84%; *p* < 0.001). Notably, 88.34% stated that there was a stressful work situation, generating PD for 71.07% of them, being this PD significantly lower for those who do not feel stress at work (15.38%; *p* < 0.001). The percentage of those who are satisfied with their work (65.92%) is higher than those who are not satisfied (34.08%), with an influence on the development of PD, which is higher among those who are not satisfied (68.29%), as compared to those who are satisfied (57.48%; *p* < 0.001).

**FIGURE 1:**
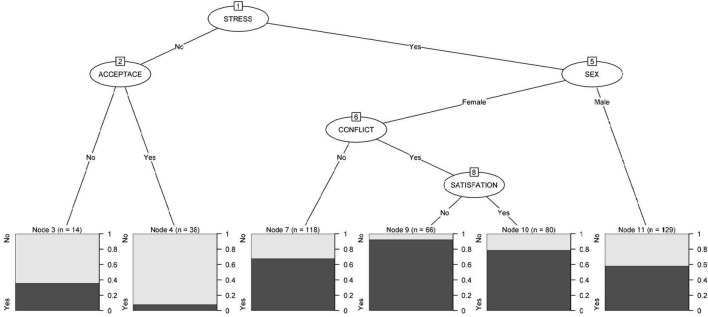
Relationship of the work environment with developing psychological distress.

Among the variables related to the work environment, work stress was shown as the most significant variable in relation to PD. No work stress was mediated by accepting the risk of infection as part of the job; among those who accept it, 7.9% have PD vs. 35.7% of those who did not. Sex, a mediating variable regarding work stress, showed a higher risk of PD among women than among men. In the case of men, the percentage of cases with PD was 58.1%. For women with work stress who believed that the situation had not increased labor disputes, the percentage of cases with PD was 67.8%, while for those who considered that their job satisfaction had increased. This was a mediating factor, distinguishing 78.8% of cases when there was satisfaction and 92.4% when there was not (Figure 1).

### Work Engagement and Development of Psychological Distress

The total mean score of the UWES-9 scale was 34.80 (SD = 10.69). Occupational health nurses and physicians with high overall engagement values had the lowest percentage of PD, i.e., 44.2%. This percentage increased in those with intermediate levels of engagement (65.2%) and was even higher in those with low engagement (76.7%; *p* < 0.001) (Table 2).

**TABLE 2:** Relationship between work environment, work engagement, and PD.

	Total		GHQ < 3		GHQ ≥ 3			
	Cases (N)	% of total	Cases (N)	% line	Cases (N)	% line	Chi-square statistic	*p*-Value
Work environment	446	100%	158	35.4%	288	64.6%	χ^2^	*p*
**Conflict**								
No	222	49.78%	105	47.30%	117	52.70%	29.28	< 0.001
Yes	224	50.22%	53	23.66%	171	76.34%		
**Risk**								
No	73	16.37%	32	43.84%	41	56.16%	4.44	0.109
Yes	373	83.63%	126	33.78%	247	66.22%		
**Acceptance**								
No	148	33.18%	44	29.73%	104	70.27%	4.88	0.087
Yes	298	66.82%	114	38.26%	184	61.74%		
**Workload**								
No	38	8.52%	24	63.16%	14	36.84%	15.84	< 0.001
Yes	408	91.48%	134	32.84%	274	67.16%		
**Stress**								
No	52	11.66%	44	84.62%	8	15.38%	64.76	< 0.001
Yes	394	88.34%	114	28.93%	280	71.07%		
**Job satisfaction**								
No	152	34.08%	33	21.71%	119	78.29%	20.91	< 0.001
Yes	294	65.92%	125	42.52%	169	57.48%		
**UWES**	446	100%	158	35.4%	288	64.6%	χ^2^	*p*
**Vigor**								
Low	111	24.9%	19	17.1%	92	82.9%	27.55	< 0.001
Intermediate	296	66.4%	116	39.2%	180	60.8%		
High	39	8.7%	23	59.0%	16	41.0%		
**Dedication**								
Low	119	26.7%	22	18.5%	97	81.5%	37.60	< 0.001
Intermediate	187	41.9%	60	32.1%	127	67.9%		
High	140	31.4%	76	54.3%	64	45.7%		
**Absorption**								
Low	115	25.8%	31	27.0%	84	73.0%	8.90	0.012
Intermediate	197	44.2%	67	34.0%	130	66.0%		
High	134	30.0%	60	44.8%	74	55.2%		
**UWES-Total**								
Low	118	26.5%	24	20.3%	94	76.7%	29.01	< 0.001
Intermediate	233	52.2%	81	34.8%	152	65.2%		
High	95	21.3%	53	55.8%	42	44.2%		

**FIGURE 2:**
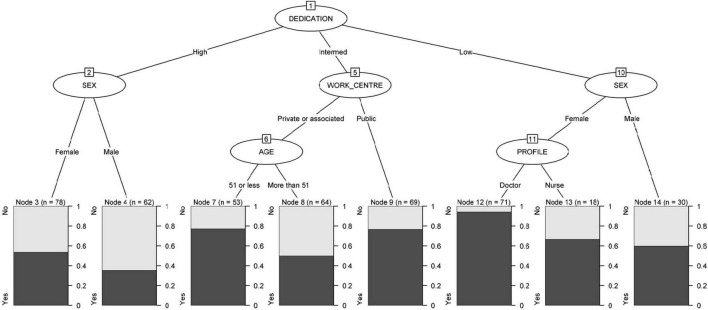
Work engagement in the development of psychological distress.

As shown in Figure 2, the degree of dedication was revealed as the most significant variable in the segmentation tree, *p* < 0.001. The participants with higher dedication had a lower percentage of PD among men (35.5%) than among women (53.8%), *p* = 0.03. With intermediate dedication, working in a private or associated center as compared to a public one was shown as a mediating factor (*p* = 0.49), as in public centers, 76.8% of professionals had PD, and in private or associated centers, depending on age, the percentage of cases with PD was 50% at ages over 51 years and 77.4% among younger professionals. In terminal nodes deriving from low dedication, 60% of men had PD, and among women, 66.7% of nurses and 94.4% of doctors had PD.

### Relationship Between Work Environment, Work Engagement, and Development of Psychological Distress

**FIGURE 3:**
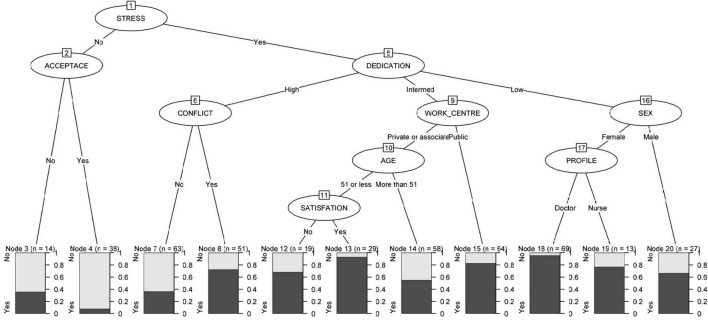
Relationship between work environment, work engagement and development of psychological distress.

Figure 3 shows how the absence of work stress and the acceptance of the risk of infection as part of the job caused PD in 7.9% of the participants, while this percentage rose to 35.7% when the risk of infection was not accepted. Regarding work stress, the dedication was revealed as a mediating variable. High dedication in a conflictive environment caused PD in 72.5% of participants, decreasing to 36.5% in the absence of conflict. Notably, 82.8% of professionals working in public work centers with average dedication presented distress. However, in private and associated centers, age was a mediating variable, finding less cases of distress among participants over 51 years of age (55.2%) and increasing in younger to 68.4% for those with a lower degree of job satisfaction in the face of a new situation and to 93.1% when this degree of satisfaction was higher. The percentage of cases with distress in professionals with low work dedication reached 66.7% in men and increased to 76.9% in the case of female nurses and 95.7% in female physicians.

## Discussion

This study aimed to describe the PD of occupational health workers and its relationship with the WE and work environment characteristics during the first months of the COVID-19 pandemic in Spain. According to the results, more than half of the participants showed PD and declared an intermediate level of WE. It was identified that a significantly higher level of PD in public sector workers than among private company workers. A relationship between WE and PD was found, and the lower WE score was related to higher PD. The dedication was the most significant dimension, and the higher scores in the dedication were related to lower PD. Regarding the work environment, those professionals who perceived conflict, stress, workload, and less job satisfaction showed more PD. Work stress was revealed as the most significant variable in relation to PD and was mediated by accepting the risk of infection as part of the job.

Occupational health professionals participating in this study showed lower PD (65.53%) than results obtained in a similar previous study conducted in Spain involving healthcare professionals (80.6%) (Gómez-Salgado et al., 2020). However, it was higher than PD identified by Rodríguez-Rey et al. (2020) (36.6%) and Odriozola-González et al. (2020) (47.5%) in Spanish general population at the same stage of the pandemic. Our results also differ from international studies, which found less PD among healthcare professionals, i.e., 41% (Krishnamoorthy et al., 2020) and 35% (Luo et al., 2020). Regarding WE, our results (34.80 SD = 10.69) are congruent with previous studies (37.93 SD = 8.52) (Buchanan et al., 2018).

Results from this study described more PD among public sector workers than in private company workers. In Spain, although the health system is mostly public, most occupational health professionals work in private companies, closer to workplaces instead of being in hospitals. Our results could be explained by the fact that the private sector involves most of the professionals of the sample. As Moretti et al. (2020) described, during the COVID-19 pandemic, workers felt less stressed since they started teleworking, thanks to avoiding traveling to the office, time flexibility, and better family life. The review conducted by Oakman et al. (2020) revealed the positive effect of working at home on mental health, reducing stress and emotional exhaustion, and improving wellbeing, quality of life, and perceived safety. However, negative effects associated with working from home during the pandemic that could increase PD have been described, such as an increased conflict between family and work and social isolation (Galanti et al., 2021). According to Xiao et al., the physical and mental wellbeing of people who work from home during the pandemic could be improved with physical exercise, healthy eating, good relationships with coworkers, adjusted working hours, control of distractions, and a suitable home environment dedicated to work (Xiao et al., 2021). The opportunity to work from home given to the participants of this study who worked at a private company may have led them to perceive less PD.

Regarding work environment, according to our results, those professionals who perceived conflict, stress, workload, and less job satisfaction claimed more PD, which is consistent with previous studies. Giorgi et al. (2020) described how work-related conditions influence moderating or worsening mental health of people during the COVID-19 pandemic. Their review pointed out that work-related stress led to mental health issues, as well as poor social support, prolonged working hours, and the perception of risk of contagion. The study conducted by Labrague and de Los Santos (2020) among frontline nurses identified that an increased level of fear of COVID-19 was associated with decreased job satisfaction and increased PD. Workload has already been identified as an influencing factor for PD suffered by healthcare workers during the COVID-19 pandemic, as well as burnout and the psychological pressure due to difficult moral decisions about care priorities in situations of emergency and shortage of resources (Sahebi et al., 2021). Additionally, Naldi et al. (2021) found an association between increased workload and moderate-to-severe symptoms of state anxiety, distress, and emotional exhaustion. It has also been identified as a major stress source for healthcare workers such as concerns about personal protective equipment, physical and emotional exhaustion, fear of being infected, and insufficient work experiences with COVID-19 (Leng et al., 2020).

The influence of perceived conflict on PD identified in this study could be related to the participants’ resilience competence, as it is considered the ability to react to adversities in a healthy, adaptive way, minimizing the psychological and physical harm (Epstein and Krasner, 2013). Many authors have pointed to resilience as a strategy for addressing the psychological impact of the pandemic on healthcare professionals (Carmassi et al., 2020; Kang et al., 2020; Pollock et al., 2020). The review conducted by Carmassi et al. (2020) described the factors to enhance the resilience and reduce the risk of adverse mental health outcomes among healthcare workers facing the COVID-19 pandemic, such as support (from family, friends, supervisors, and colleagues), training, prompt work organization, and good coping strategies. Training programs aimed at building resilience can improve self-confidence and psychological skills and can encourage occupational health professionals to cope with dramatic situations (Kang et al., 2020).

In this study, a relationship between WE and PD was found, and high engagement values predicted lower levels of PD; dedication was the most significant dimension, and the higher scores in the dedication were related to lower PD. These results are in line with previous studies (Ruiz-Frutos et al., 2021) in which dedication was also the most valued dimension by healthcare professionals (Gómez-Salgado et al., 2020). Our findings are expectable, as dedication refers to the significance of the work, to feel enthusiastic, proud, and inspired by the work done (Schaufeli et al., 2002), also keeping a positive attitude (Chew et al., 2020). As Carmassi et al. (2020) identified, support from supervisors and colleagues can play a protective role for mental health of healthcare workers when facing the COVID-19 outbreak.

When interpreting results related to PD, the effect of vaccination on workers should be considered. Vaccination against COVID-19 has been identified to improve the physical and mental wellbeing of workers, by reducing anxiety and improving mood and comfort in job performance (Haddaden et al., 2021). According to the study by Karayürek et al. (2021), vaccination had a positive effect on reducing the fear and anxiety levels of health professionals. However, reluctance on the part of some sectors of the population toward the vaccine has been described, based on fear of the side effects of the vaccine, doubts about its safety, the availability of scant or contradictory information about them, or beliefs in conspiracy theories (Akarsu et al., 2021; Di Gennaro et al., 2021; El-Elimat et al., 2021).

There is a need for those responsible for managing health of the workers to be aware of the status and factors associated with the mental health and work attitudes of employees during the COVID-19 pandemic, such as WE and job satisfaction (Song et al., 2020). Thus, work organizations need to address emergent changes in daily work practices, such as virtual teamwork, leadership, and management, or even social distancing (Gómez-Salgado et al., 2021). These problems can occur in the context of home teleworking, raising concerns about work-family issues (Kniffin et al., 2021). Thus, alongside the public health measures, an appropriate occupational health response is also necessary. It is essential to maintain the health and psychological wellbeing of occupational health professionals so they can fulfill their mission.

As a limitation of this study, it should be acknowledged the convenience sample that could limit the generalization of the results and the non-inclusion of specific resilience measurement instruments in the survey, despite being an important factor in the generation of PD. The sample distribution is not equitable, more female participants, physicians, and from the private sector, which could induce some bias. In addition, due to the cross-sectional design of the study, although the relationship between variables has been identified, it prevents describing changes in variables over time, as well as the direction of relationships. Another limitation is the scarcity of articles focused on the psychological health of occupational health workers. This has made it difficult to compare the results, which have been contrasted with studies on health professionals, being able to introduce certain biases as they are a group of health professionals with differentiated and specific competencies and responsibilities. However, having carried out the study in the first phase of the pandemic will allow us to know the variation with respect to later phases or future pandemics.

## Conclusion

The COVID-19 has made an impact in the workplace affecting all workers, so preventive and safety measures have to be undertaken so as to be able to adapt to the new requirements of the pandemic, such as protective measures against PD. In our study, 65.53% of the occupational health professionals who participated had PD (GHQ ≥ 3). No significant differences were found between physicians and nurses; however, PD was higher among women and public sector workers. Variables that facilitate developing PD were work stress, workload, the presence of labor conflict, and less job satisfaction. All the dimensions of WE acted as mediators in PD.

The results of this study could help to understand the vulnerable situation of occupational health professionals as a consequence of the pandemic with respect to mental health. Interventions are needed to alleviate the PD suffered by most of these workers, especially in the public sector. According to our results, they should help to deal with the workload, conflict, and an increase in job satisfaction. In light of our results, organizational and management strategies that promote WE are suggested, given the effect on mental health identified in this study. These measures could have an impact on the psychological wellbeing of workers by increasing their ability to cope and resilience.

## Data Availability Statement

The raw data supporting the conclusions of this article will be made available by the authors, without undue reservation.

## Ethics Statement

The studies involving human participants were reviewed and approved by Research Ethics Committee of Huelva. The patients/participants provided their written informed consent to participate in this study.

## Author Contributions

CR-F, MO-M, MR-M, JC-M, and JG-S: conceptualization, data curation, formal analysis, investigation, methodology, project administration, resources, software, supervision, validation, visualization, writing—original draft, and writing—review and editing. GS-T: conceptualization, data curation, formal analysis, investigation, methodology, resources, software, supervision, validation, visualization, writing—original draft, and writing—review and editing. RA-C: conceptualization, data curation, formal analysis, funding acquisition, investigation, methodology, resources, software, supervision, validation, visualization, writing—original draft, and writing—review and editing. All authors contributed to the article and approved the submitted version.

## Conflict of Interest

The authors declare that the research was conducted in the absence of any commercial or financial relationships that could be construed as a potential conflict of interest.

## Publisher’s Note

All claims expressed in this article are solely those of the authors and do not necessarily represent those of their affiliated organizations, or those of the publisher, the editors and the reviewers. Any product that may be evaluated in this article, or claim that may be made by its manufacturer, is not guaranteed or endorsed by the publisher.
